# Genome-scale metabolic network model and phenome of solvent-tolerant *Pseudomonas putida* S12

**DOI:** 10.1186/s12864-023-09940-y

**Published:** 2024-01-16

**Authors:** Sol Han, Dohyeon Kim, Youngshin Kim, Sung Ho Yoon

**Affiliations:** Department of Bioscience and Biotechnology, Konkuk University, Seoul, 05029 Republic of Korea

**Keywords:** *Pseudomonas putida* S12, Metabolic network model, Genome, Phenome, Flux balance analysis

## Abstract

**Background:**

*Pseudomonas putida* S12 is a gram-negative bacterium renowned for its high tolerance to organic solvents and metabolic versatility, making it attractive for various applications, including bioremediation and the production of aromatic compounds, bioplastics, biofuels, and value-added compounds. However, a metabolic model of S12 has yet to be developed.

**Results:**

In this study, we present a comprehensive and highly curated genome-scale metabolic network model of S12 (iSH1474), containing 1,474 genes, 1,436 unique metabolites, and 2,938 metabolic reactions. The model was constructed by leveraging existing metabolic models and conducting comparative analyses of genomes and phenomes. Approximately 2,000 different phenotypes were measured for S12 and its closely related KT2440 strain under various nutritional and environmental conditions. These phenotypic data, combined with the reported experimental data, were used to refine and validate the reconstruction. Model predictions quantitatively agreed well with in vivo flux measurements and the batch cultivation of S12, which demonstrated that iSH1474 accurately represents the metabolic capabilities of S12. Furthermore, the model was simulated to investigate the maximum theoretical metabolic capacity of S12 growing on toxic organic solvents.

**Conclusions:**

iSH1474 represents a significant advancement in our understanding of the cellular metabolism of *P. putida* S12. The combined results of metabolic simulation and comparative genome and phenome analyses identified the genetic and metabolic determinants of the characteristic phenotypes of S12. This study could accelerate the development of this versatile organism as an efficient cell factory for various biotechnological applications.

**Supplementary Information:**

The online version contains supplementary material available at 10.1186/s12864-023-09940-y.

## Background

*Pseudomonas putida* is a gram-negative gamma-proteobacterium widely distributed in soil and water environments. *P. putida* has a broad metabolic range and can tolerate and degrade various organic compounds [1, 2]. Therefore, it has been extensively used in bioremediation to remove organic pollutants from contaminated sites. This bacterium exhibits rapid growth, metabolic versatility, and inherent robustness to physicochemical stresses, making it an ideal candidate for producing biofuels, bioplastics, and other industrial products using renewable feedstocks [1, 3].

*P. putida* S12 is exceptionally tolerant to various organic solvents and aromatic compounds that are toxic to most bacteria [4]. It was first isolated using styrene as the sole carbon source [5], and it can grow on supersaturated concentrations of styrene, octanol, or heptanol as the sole carbon source [6]. Due to its high solvent tolerance and versatile metabolism, S12 has been used in the synthesis of organic compounds such as phenol [7], *p*-hydroxystyrene [8], *p*-hydroxybenzoate [9], and 2,5-furandicarboxylic acid [10]. The complete genome of S12 consists of a chromosome of 5,798,534 bp and a megaplasmid pTTS12 of 583,900 bp [11]. The S12 is phylogenetically closest to the plant growth-promoting rhizobacterium *P. putida* BIRD-1 [12], which harbors a chromosome without plasmid [13]. The chromosome size of S12 is comparable to that of *P. putida* BIRD-1 (5,731,541 bp) [12], and is less than that of the best characterized saprophytic *P. putida* KT2440 (6,181,873 bp) lacking a plasmid [14]. The pTTS12 represents the largest megaplasmid identified in *P. putida*, encoding many unique features such as styrene catabolism, the solvent-resistance pump and resistance to heavy metals [11].

A genome-scale metabolic network model (GEM) of an organism is a computational representation of the entire metabolism that integrates genome annotation, biochemical information, and experimental data to generate a comprehensive inventory of the metabolic pathways and reactions occurring in the organism [15]. GEMs have been developed for diverse organisms [16] and have been used to understand metabolic capabilities and genotype–phenotype relationships and to design metabolically engineered strains for various biotechnological applications [17–19]. Strain-specific metabolic models have been reconstructed for non-pathogenic and pathogenic *Pseudomonas* genera. These include opportunistic human pathogenic *P. aeruginosa* strains PAO1 [20] and PA14 [20–22], endophytic *P. stutzeri* A1501 [23], and *P. fluorescens* SBW25 [24]. Among the *P. putida* strains, GEMs have been developed exclusively for KT2440 [25–28], while a GEM for S12 has yet to be reported.

In this study, we developed a comprehensive and highly curated GEM for S12 by leveraging the published GEMs of *P. putida* KT2440 and *P. aeruginosa* PAO1 and performing comparative genome and phenome analyses. Phenotype microarray (PM) tests was performed for S12 and KT2440 to validate and update the model. The resulting GEM was simulated to explore the metabolic features of S12.

## Results

### Construction of a metabolic network model

**Fig. 1:**
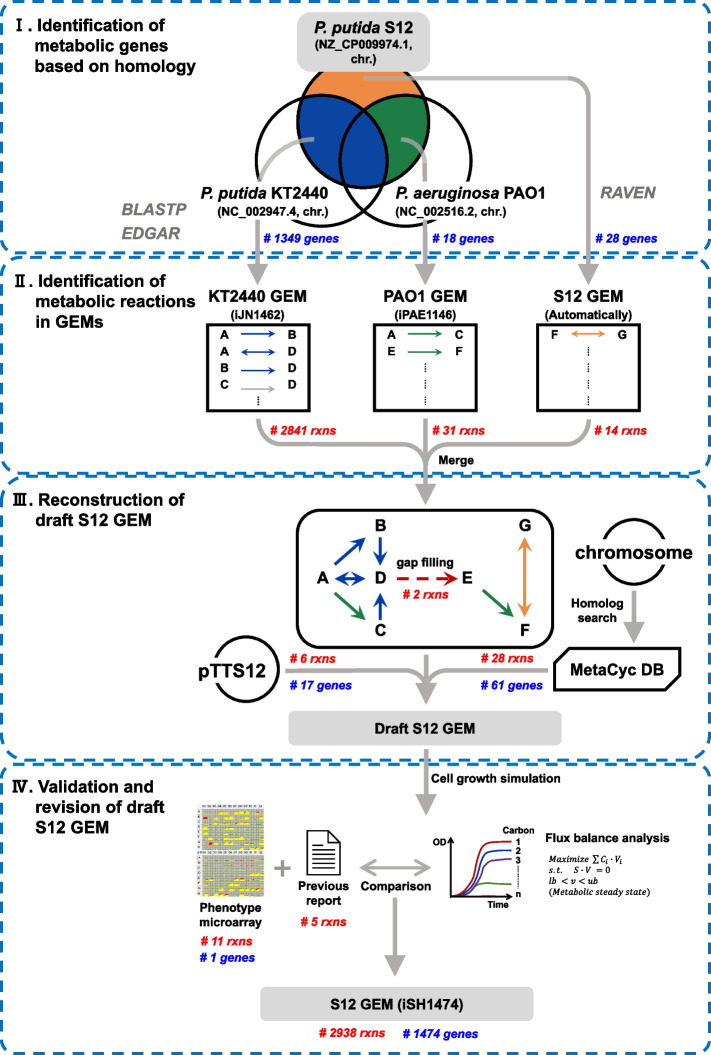
Workflow of the reconstruction of the metabolic network model of *P. putida* S12. The numbers of metabolic genes and reactions identified in each step are shown

Draft S12 GEM reconstruction was initiated by identifying homologs of S12 in genes contained in the published GEMs of *P. putida* KT2440 (iJN1462) [25] and *P. aeruginosa* PAO1 (iPAE1146) [20] (Fig. 1). *P. putida* KT2440 was chosen as it is a well-characterized model strain of *P. putida* [1] and it is closely related to S12. *P. aeruginosa* PAO1 has a high-quality genome annotation through the *Pseudomonas* community annotation project [29]. Homology searches were performed using the genomes of KT2440 [14] and PAO1 [30] against the S12 chromosome [11]. The EDGAR server (version 3.0) [31] was used to identify additional S12 homologs in KT2440 and PAO1. As a result of these homology searches, 1,349 metabolic genes of S12 were observed to have homologs in the KT2440 GEM, and 2,841 associated metabolic reactions. Eighteen homologs of S12 were identified in the PAO1 GEM, which were not observed in the KT2440 GEM, and their 31 associated metabolic reactions were retrieved.

Automated metabolic reconstruction of S12 using the RAVEN toolbox [32] identified 28 metabolic genes and 14 metabolic reactions that were absent in iJN1462 and iPAE1146 (Table S1). Homology searches were performed using the S12 chromosome against the NCBI non-redundant (NR) database, which yielded 61 additional metabolic genes with potential metabolic roles. Twenty-eight associated metabolic reactions were retrieved from the MetaCyc database [33]. Seventeen metabolic genes and six reactions encoded by the megaplasmid pTTS12 were retrieved from a previous study [34].

The reconstruction was manually gap-filled. The initial version of the model was constructed by gathering reactions and metabolites from iJN1462, iPAE1146, and the automated metabolic reconstruction of S12. When the FBA simulation was run using the model, it showed no growth, indicating the absence of certain reaction(s) required for generating specific component(s) in the biomass equation. Therefore, the reactions of iJN1462 and iPAE1146, which were previously left out due to the absence of an associated S12 homolog, were sequentially added to the model, however, these additions did not lead to the simulated growth. Iteratively adding pairs of reactions identified two reactions leading to the simulated growth. They were NADS1 and CLt3_2pp, which are mediated by ammonia-dependent NAD( +) synthetase and chloride channel protein, respectively, contained in iJN1462. NADS1 is associated with the S12 gene that didn’t satisfy the criteria for the homolog search, while CLt3_2pp is not associated with any S12 gene. Additionally, 350 exchange reactions for external metabolites were included in the model.

For functionality of the model, the model includes artificial reactions of sink and demand reactions present in iJN1462 [25]. To remove dead end metabolites [35], the model contains 31 demand reactions, of which 24 are related to various forms of polyhydroxyalkanoate (PHA). *Pseudomonas* species is well-known for storing PHAs as a reverse for carbon and energy under unbalanced growth conditions [36]. As PHAs are accumulated intracellularly, they become a dead end metabolite that needs to be removed through demand reactions. As PHAs are not a part of the biomass equation, in silico growth does not require PHA synthesis. Inclusion of the demand reactions for PHAs are primarily to represent the characteristics of the *P. putida*. The model also contains two sink reactions to generate metabolites whose biosynthetic pathways are not fully known; "sink_PHAg," which supplies the PHA granule needed for PHA biosynthesis, and "sink_pqqA," which provides the initial peptide required for biosynthesis of pyrroloquinoline-quinone (PQQ). Based on PM tests and previous reports, the model was revised, and 16 additional metabolic reactions were incorporated (see below).

To provide annotation information of genes, reactions, and metabolites in the sbml file of the model, web links to public databases were generated by COBRApy [37]. To annotate the genes, UniProt [38] annotations were obtained by searching the MetaCyc database. We included gene annotation links for refseq_locus_tag, ncbigi, and refseq_name, even though they are currently inaccessible, because this information was sourced from the RefSeq GenBank file of S12. The nomenclature of the metabolic reactions and metabolites followed the BiGG model database [16]. The resulting S12 GEM (iSH1474) is available in Excel (Table S2) and SBML formats (File S1).

### Generation of the biomass equation

The biomass equation of iSH1474 was generated based on the KT2440 GEM (iJN1462) [25] by adjusting S12-specific factors (Table S3). Compositions of amino acids, deoxynucleotides (dNTPs), and nucleotides (NTPs) were estimated using the genome sequence of S12, according to Thiele and Palsson [35]. To calculate the non-growth-associated maintenance energy (NGAM), an experimental plot of the growth rate versus glucose uptake rate was generated using the maintenance coefficient and maximum growth yield, which were determined from glucose-limited chemostat cultures of *P. putida* S12 growing aerobically on a minimal medium [39]. NGAM value of 1.67 mmol ATP/gDCW/h was calculated from the y-intercept of the experimental plot (Fig. S1). While keeping the NGAM value fixed, flux balance analysis (FBA) was performed by varying the value of the growth-associated maintenance energy (GAM) in the biomass equation to determine the value that provided the closest fit to the experimental plot (42.31 mmol ATP/gDCW) [35].

### Metabolic pathways featured in *P. putida* S12

**Fig. 2:**
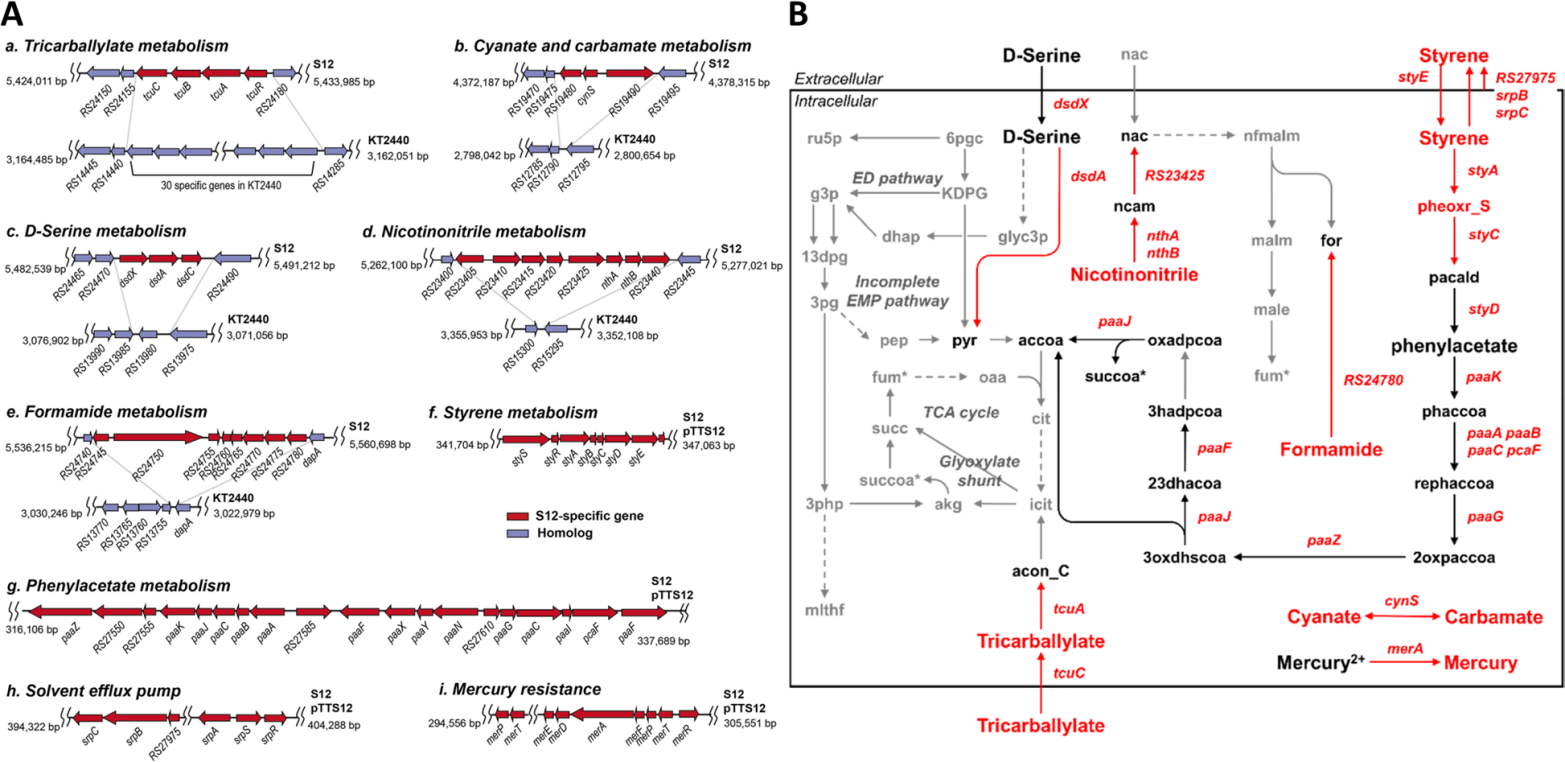
Metabolic gene clusters and their corresponding metabolic pathways present only in S12 compared to KT2440. **A** Metabolic gene clusters. (*a-e*) Gene clusters in the S12 chromosome: catabolism of tricarballylate (*a*), cynate and carbamate (*b*), D-serine (*c*), nicotinonitrile (*d*), and formamide (*e*). (*f-i*) Gene clusters in the pTTS12 plasmid: catabolism of styrene (*f*), phenylacetate (*g*), solvent efflux pump (*h*), and mercury (*i*). For clarity, the locus tags RPPX_ and PP_ were removed from the locus names of S12 and KT2440, respectively. **B** Metabolic pathways. Among the reactions mediated by the gene clusters shown in panel A, those specific to S12 are colored in red, and those common to S12 and KT2440 are shown in black. Reactions not associated with the S12-specific gene clusters are colored in grey

To identify the metabolic gene clusters specific to S12, the S12 genome was compared to the KT2440 genome. Fourteen metabolic gene clusters containing at least three genes were observed exclusively in S12. In contrast, only five metabolic gene clusters were observed in KT2440 (Table S4). On the S12 chromosome, these unique gene clusters encode enzymes involved in the catabolism of tricarballylate, cynate/carbamate, D-serine, nicotinonitrile, and formamide (Fig. 2A). One notable cluster was a four-membered gene cluster resembling the tricarballylate utilization locus (*tcuRABC*) of *Salmonella enterica* serovar Typhimurium [40, 41]. Within this locus, RPPX_RS24175, RPPX_RS24170, and RPPX_RS24165 showed homology to the regulatory protein (TcuR), tricarballylate dehydrogenase (TcuA), and a putative electron shuttle protein (TcuB), respectively. However, the gene RPPX_RS24160, annotated as a citrate symporter, showed less homology with the tricarballylate transporter TcuC (21% amino acid identity). This locus in S12 replaced 30 genes in the KT2440 genome, including ABC transporters and KT2440-specific genes, such as FMN reductase and dimethyl sulfone monooxygenase. Regarding D-serine metabolism, both S12 and KT2440 possess the D-amino acid dehydrogenase (DadA), which converts D-serine into β-hydroxy pyruvate. However, only S12 contains a D-serine utilization locus (*dsdXAC*) that includes genes for the D-serine transporter (DsdX), D-serine deaminase (DsdA), and LysR-type regulator (DsdC) [42, 43]. In the megaplasmid pTTS12, several gene clusters were identified in S12, including those involved in styrene degradation (encoded by *styABCD*), phenylacetate degradation (*paa* gene cluster), solvent efflux pumps (*srp* genes), and resistance to heavy metals such as mercury, tellurite, and chromate (*mer* genes) [34]. The *paa* gene cluster, which lacks *paaN* and has a different gene order, was observed on the chromosomes of both S12 and KT2440. All these unique gene clusters observed in *P. putida* S12 and associated metabolic reactions were added to the S12 GEM (Fig. 2B).

### Phenome analysis

**Fig. 3:**
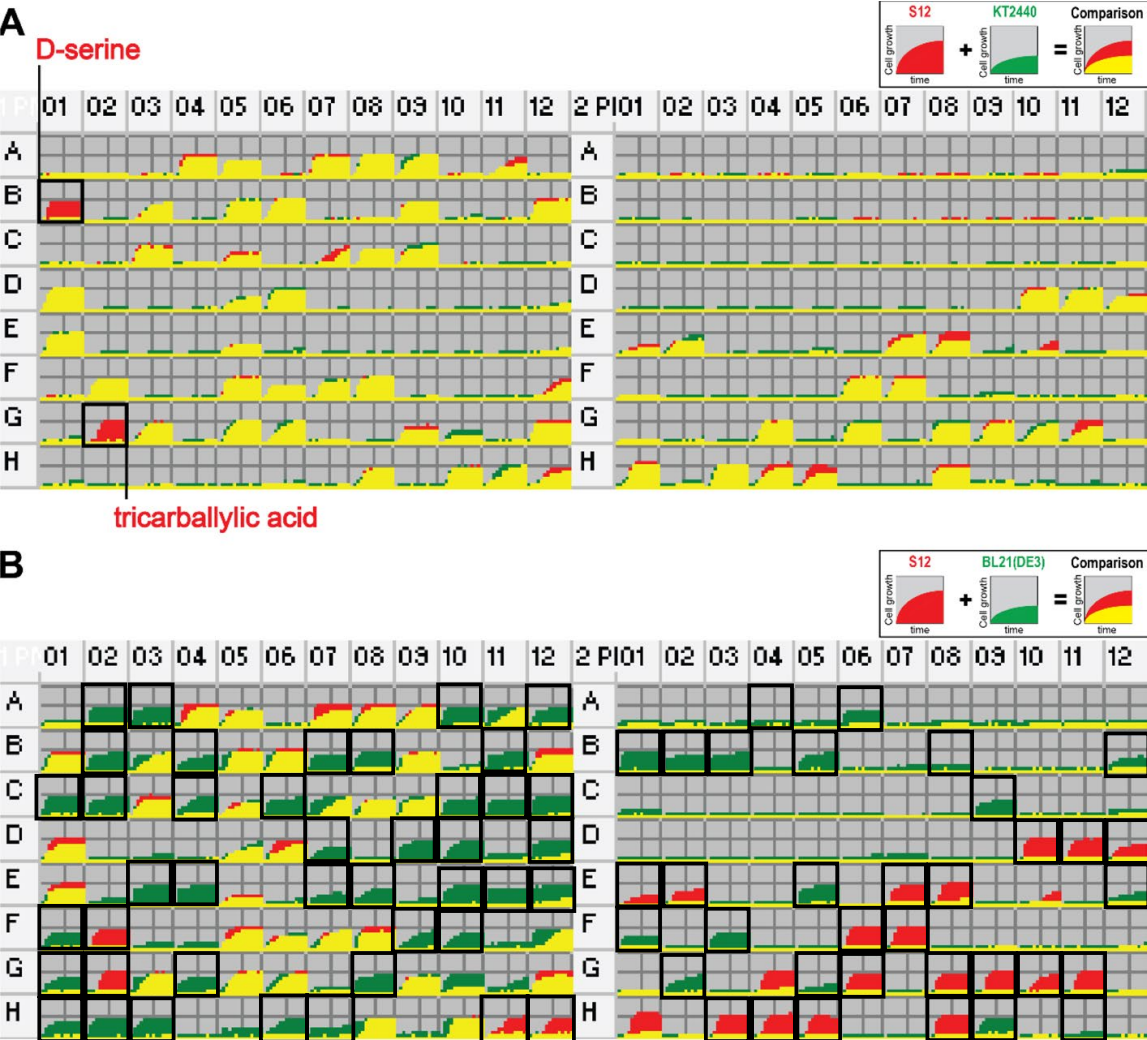
Comparison of carbon source utilization of *P. putida* S12 with other strains. **A** *P. putida* S12 vs. *P. putida* KT2440. **B** *P. putida* S12 vs. *E. coli* BL21(DE3). Growth curves in all the cells of PM1 and PM2 are colored red for faster growth of S12, green for faster growth of KT2440 or BL21(DE3), and yellow for similar growth of both strains. PM wells showing strain-specific cell growth are boxed. The complete list of PM results is available in Table S2 and Fig. S2

The PM tests assess phenotypic growth on approximately 2,000 substrates using microtiter plates (PM1 to PM20) containing many different carbon, nitrogen, and phosphorus sources and other compounds [44]. To investigate the metabolic behavior and strain-specific metabolic capacity of S12, PM tests were conducted on both S12 and KT2440 (Fig. S2A and Table S5). S12 and KT2440 exhibited similar growth patterns on most digestible substrates (PM1 to PM8), suggesting that both strains possessed comparable metabolic capabilities for utilizing these substrates. Among the 190 carbon sources tested (PM1 and PM2), S12 and KT2440 exhibited aerobic growth on 58 and 56 substrates, respectively (Fig. 3A). S12 grew on two carbon sources, tricarballylic acid and D-serine, which KT2440 could not utilize. This can be attributed to the presence of gene clusters (*tcuRABC* and *dsdXAC*) in S12, which are involved in the utilization of tricarballylate and D-serine (Fig. 2). Among the 59 phosphorus sources tested (PM4), S12 and KT2440 showed the same growth patterns, with 37 sources growing and 22 sources not growing.

Both S12 and KT2400 could grow regardless of the type of inhibitory compounds present in PM11–PM20, which included antibiotics, antimetabolites, and other inhibitors. However, certain inhibitory compounds prevented growth at high concentrations. S12 exhibited additional growth in 19 wells containing high concentrations of specific compounds compared with KT2440. These compounds included 3-amino-1,2,4-triazole, acriflavine, boric acid, cefsulodin, cobalt(II) chloride, coumarin, dodecyltrimethylammonium bromide, dodine, doxycycline, enoxacin, hexaminecobalt(III) chloride, kanamycin, minocycline, potassium chromate, potassium tellurite, protamine sulfate, sodium dichromate, sodium metaborate, and tobramycin. In contrast, KT2440 showed growth at higher concentrations of eight different chemicals: alexidine, cadmium chloride, crystal violet, methyltrioctylammonium chloride, rifampicin, ruthenium red, sulfanilamide, and tetrazolium violet. These results indicate that S12 and KT2440 resist a broad range of inhibitory compounds. Their tolerance can vary depending on the specific chemical compound.

To further explore the metabolic capacity and tolerance of S12, its PM data were further compared to the PM data of another popular industrial host, *E. coli* BL21(DE3) [45] (Fig. S2B and Table S5). Regarding carbon sources, S12 and BL21(DE3) showed substantial differences in their ability to utilize different substrates for growth. S12 grew on 58 carbon sources, while BL21(DE3) grew on a larger number of carbon sources (91 ea) (Fig. 3B). Only 36 carbon sources were utilized by both strains. S12 exhibited growth on 22 carbon sources that BL21(DE3) could not utilize, including citric acid and β-phenylethylamine. Conversely, BL21(DE3) grew on 55 carbon sources, including L-arabinose and D-xylose, which S12 did not utilize. S12 could grow under aberrant osmolarity and pH conditions, whereas BL21(DE3) did not show such tolerance, suggesting that S12 has a higher tolerance to variations in osmolarity and pH. Furthermore, compared to that in BL21(DE3), S12 displayed greater tolerance to a wider range of toxic chemical compounds. S12 grew in the presence of 17 chemicals (2,2′-dipyridyl, captan, carbenicillin, ciprofloxacin, cobalt(II) chloride, enoxacin, furaltadone, nalidixic acid, nitrofurazone, norfloxacin, ornidazole, potassium chromate, protamine sulfate, sulfisoxazole, tinidazole, tolylfluanid, and trimethoprim), while BL21(DE3) did not. This divergence in tolerance to toxic compounds further emphasizes the different metabolic capabilities and adaptabilities of the two gamma-proteobacteria. The observed differences in nutrient utilization and tolerance capacities between S12 and BL21(DE3) imply that these two gamma-proteobacteria have distinct metabolic profiles, likely resulting from variations in their genetic makeup, environmental adaptations, or evolutionary histories.

### Model refinement and validation

The draft model was revised by comparing its predictions with the PM data. The simulated growth did not agree with the experimental growth for 15 of the 190 carbon sources tested. False positives were observed among these disagreements, where the model predicted growth. However, the PM tests showed non-growth for utilizing seven carbon sources (2,3-butanediol, acetoacetic acid, glycine, L-homoserine, L-phenylalanine, L-threonine, and uridine). S12 did not grow on D-ribose in the PM test; however, its growth on D-ribose has been reported to occur during prolonged cultivation after 240 h [46], which agreed with the model prediction. The exchange reactions for these carbon sources were removed from the model to align with the experimental non-growth observations. Conversely, there were false negatives where the PM tests showed growth. However, the model predicted non-growth for eight carbon sources (tween 20, tween 40, bromosuccinic acid, mono-methylsuccinate, D-ribono-1,4-lactone, butyric acid, L-alanyl-glycine, and tricarballylic acid).

The MetaCyc database [33] was searched for transport reactions for these carbon sources, and then their associated genes were BLASTP-searched to find homologs in the S12 genome. The S12 genome was found to possess genes associated with utilization of three carbon sources (butyric acid, L-alanyl-glycine, and tricarballylic acid). The remaining five carbon sources were considered unusable due to the absence of genes associated with their catabolic or transport reactions. To transport the three carbon sources, exchange reactions (from the extracellular environment to the periplasm) and transport reactions (from the periplasm to the cytosol) were added to the model (Table S1). For tricarballylate utilization, the gene RPPX_RS24160 was considered the tricarballylate transporter (TcuC), and its associated reaction (TCBt2pp) was added to the model. This assignment was based on the essential role of TcuC in *S. enterica* growth using tricarballylate as the sole carbon source [41]. The transporter reaction for L-alanyl-glycine (ALAGLYabcpp) was included, and its gene-protein-reaction rule was assigned to existing aminopeptide transporter genes. Although these genes showed high protein sequence similarity to genes in the MetaCyc database (> 94% identity and 100% coverage), however, were not included in the automatic reconstruction using RAVEM for unknown reason. The transporter reaction for butyric acid (BUTt2rpp) was added, and its gene-protein-reaction rule was assigned to RPPX_RS22845, which showed homology with the *E. coli atoE* gene (33% identity and 95% coverage) encoding a short-chain fatty acid transporter gene.

Regarding utilizing organic solvents as the sole carbon source, it was reported that S12 could grow on three solvents (heptanoate, octanol, and styrene). In contrast, it did not grow on ten other solvents (benzene, cyclohexane, decane, dimethylphthalate, ethylbenzene, fluorobenzene, hexane, *p*-xylene, propylbenzene, and toluene) [6]. The model predictions for the three organic solvents (octanol, styrene, and benzene) did not agree with the experimental results. To reflect these discrepancies, the artificial transport reactions for octanol (OCTANOLpp and OCTANOLtex) and styrene (styrenepp) were added to the model, based on the experimental evidence [5, 6].

**Fig. 4:**
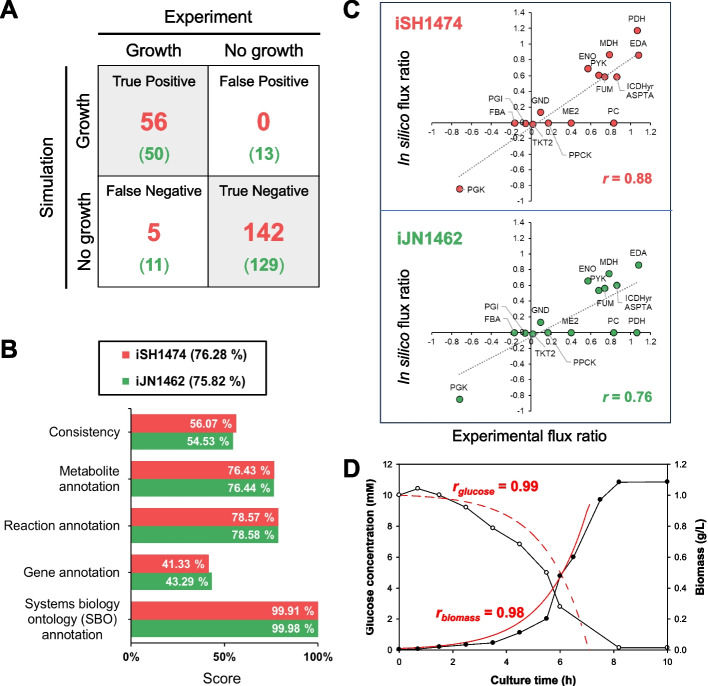
Model validation. **A** Comparison of growth predictions using iSH1474 (Simulation) and experimental growths on 190 carbon sources contained in the phenotype microarray and 13 organic compounds (Experiment). The number in the parenthesis denotes the number of growth predictions using iJN1462. **B** Comparison of MEMOTE reports of iSH1474 (S12) and iJN1462 (KT2440). **C** Comparison of flux distribution in the central carbon metabolism of S12 from in vivo measurements and in silico predictions. Reported experimental flux values (on the x-axis) are compared with the predicted fluxes (y-axis) using iSH1474 (S12) and iJN1462 (KT2440). The flux values are normalized to the experimental glucose uptake rate. **D** Dynamic flux balance analysis using iSH1474 for time profiles of biomass and glucose concentration in aerobic batch growth of S12 on glucose as the sole carbon source. Black and white circles denote experimental data of biomass and glucose concentration in the culture medium [39], respectively. Solid and dashed red lines represent the model predictions for biomass and glucose concentration, respectively

After conducting a thorough manual curation process, the final version of the S12 GEM (named iSH1474) consisted of 1,474 genes, 1,436 unique metabolites, and 2,938 metabolic reactions (Table 1). To assess the predictive accuracy of the model, the simulated growths were compared with the experimental results for 203 carbon sources (190 carbon sources from the PM test (Table S6) and 13 organic solvents). The growth predictions using iSH1474 qualitatively agreed well with the experimental observations for 197 carbon sources, with a predictive accuracy of 97.5% (Fig. 4A). This contrasts with the predictive accuracy of 88.2% achieved by the KT2440 iJN1462 model when simulating the same experimental data, demonstrating the significance of iSH1474 model in accurately predicting S12 phenotype.

**Table 1:** Comparison of the metabolic network model of *P. putida* strain S12 (iSH1474) and KT2440 (iJN1462)

Metabolic Model	iSH1474 (S12)	iJN1462 (KT2440)
Genes	1474	1462
Metabolic reactions	2938	2929
Enzymatic reactions	1723	1720
Transport reactions	834	827
Exchange reactions	350	351
Demand reactions	31	31
Gene-reaction association	2938	2929
Gene-associated reactions	2067	2089
Not gene-associated reactions	831	800
Spontaneous reactions	40	40
Metabolites	2167	2155
Cytoplasmic	1339	1341
Periplasmic	471	465
Extracellular	357	349
Unique metabolites	1436	1434

The overall quality of iSH1474 was evaluated using MEMOTE, a metabolic model-testing tool. MEMOTE evaluates different aspects of a metabolic model, and the final score is the weighted sum of all individual test results [47]. The subsections “Consistency” and “SBO annotation” have higher weights of 3 and 2, respectively, as they are crucial for the overall quality and utility of the model. The other subsections covering annotations of metabolites, reactions and genes have lower weights of 1. The iSH1474 model received the individual scores for consistency (56.1%), systems biology ontology (99.9%), and annotation of genes (41.3%), reactions (78.6%), and metabolites (76.4%). The final score (76.3%) was higher than that of the high-quality KT2440 iJN1462 model (75.8%) (Fig. 4B). The high quality score indicates that iSH1474 is a reliable and accurate representation of S12's metabolic capabilities.

### Comparison of quantitative simulations with experimental data

To further validate the quality of iSH1474, the predicted fluxes were compared with the experimentally reported fluxes obtained from ^13^C-based metabolic flux analysis (^13^C-MFA) of S12 grown in glucose-containing minimal medium [48]. The reported S12 fluxes showed a higher correlation with the iSH1474-based fluxes (Pearson correlation coefficient *r* = 0.88), compared to the iJN1462-based fluxes (*r* = 0.76) (Fig. 4C and Fig. S3). Both GEMs failed to predict flux through pyruvate shunt which converts malate to pyruvate by the malic enzyme (ME2) and then to oxaloacetate by pyruvate carboxylase (PC). The pyruvate shunt is the characteristics of *P. putida* to generate a high level of NADPH crucial for tolerance to oxidative stress [48, 49]. However, as this pathway is energy inefficient and the carbon flux control at the PEP-pyruvate-oxaloacetate node is complex [50, 51], FBA cannot predict the accurate flux distribution through this pathway [25]. Despite this intrinsic limitation of the FBA, the higher predictive accuracy of iSH1474 over iJN1462 highlights iSH1474 is well suited to predict flux distribution in the central carbon metabolism of S12.

To assess if iSH1474 can be used to accurately simulate the metabolic behavior of S12 in response to the environmental change over time, dynamic flux balance analysis (dFBA) [52, 53] was performed for the batch cultivation of S12 aerobically growing in a minimal medium supplemented with glucose as the sole carbon source [39] (Fig. 4D). The simulation results qualitatively aligned well with the experimental data for both biomass (*r* = 0.99) and glucose concentration (*r* = 0.98). This high correlation indicates that iSH1474 can effectively predict the metabolic adjustments of S12 under varying culture conditions.

### Gene essentiality analysis

Identifying essential genes provides valuable insights into the key components and metabolic pathways vital for sustaining growth in an organism. To analyze the candidate essential genes in S12 growing aerobically in a minimal glucose medium, simulations of single-gene deletions were performed using iSH1474. The predicted 256 essential genes of S12 (Table S7) were compared with the 262 essential genes of KT2440 predicted using iJN1462. S12 and KT2440 shared the predicted 249 essential genes (Fig. S4A), indicating a significant overlap of essential genes in these strains. However, seven genes were considered essential only in the S12 GEM. These genes included RPPX_RS01240, RPPX_RS10345 (*dapF*), RPPX_RS11450 (*hisI*), RPPX_RS14405, RPPX_RS15620, RPPX_RS23635 (*hemB*), and RPPX_RS17065 (*tmk*) (Fig. S4B). The metabolic reactions related to these genes, except for *tmk*, were associated with several genes in KT2440, suggesting that the loss of any of these genes in KT2440 could be compensated for by the presence of another gene with overlapping functions. The gene *tmk*, which encodes thymidylate kinase, was only present in S12, which is homologous to a gene in *P. aeruginosa* PAO1. Conversely, 13 genes were predicted to be essential only in the KT2440 GEM. One gene (PP_3959) was absent in S12. Four other genes in KT2440 (PP_0321, PP_0527, PP_1995, and PP_4862) corresponded to the S12 genes, whose associated reactions could be carried out by alternative reactions in S12. The metabolic reactions related to the remaining eight genes are associated with several genes in S12. This gene essentiality information provides valuable insights into the industrial applications of S12, such as the design of metabolically engineered strains.

### Simulation of the utilization of organic solvents

**Fig. 5:**
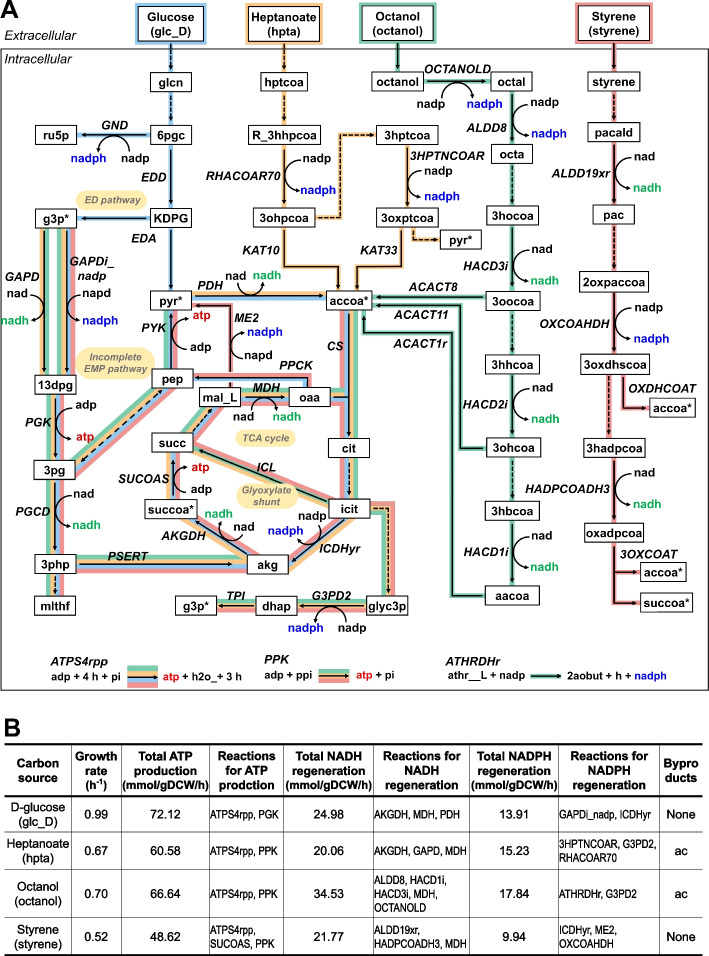
Model predictions of growth capability of S12 growing aerobically on heptanoate, octanol, styrene, and glucose. **A** The simulated catabolic routes. Metabolites are in the box, and metabolic reactions are in italic bold. Arrows denote directions of the predicted metabolic fluxes, and dashed arrows indicate the multi-step reaction. Metabolites colored above the arrows indicate cofactors of ADP, ATP, NAD(P), and NAD(P)H. The metabolites (acetyl-CoA [accoa], glyceraldehyde 3-phosphate [g3p], succinyl-CoA [succoa], and pyruvate [pyr]) duplicated on the map are labeled with an asterisk. Each catabolic route is indicated by a different color. **B** Predicted growth rate, ATP production, and NAD(P)H regeneration according to the carbon source utilized. “Reactions for ATP production” and “Reactions for NAD(P)H regeneration” are reactions responsible for > 90% of total ATP production and total NAD(P)H regeneration, respectively, in descending order. For flux balance analysis, the upper limits of oxygen and each carbon source were set to 18.5 and 10 mmol/gDCW/h, respectively. Abbreviations are given in Table S2

Following the completion of the metabolic model construction and subsequent model validation, we used iSH1474 to explore metabolic features of S12 that had not been previously investigated. To investigate the maximum theoretical metabolic capacity of S12 growing on toxic organic solvents, iSH1474 was simulated for its aerobic utilization of three organic solvents (heptanoate, octanol, and styrene) as well as glucose for comparison. The simulated catabolic routes for these carbon sources revealed distinct metabolic pathways (Fig. 5A). All these are converted into acetyl-CoA to enter the tricarboxylic acid (TCA) cycle. For glucose and styrene, the carbon flux was divided between the full TCA cycle and the glyoxylate shunt at a ratio of 1:1.1. In contrast, a significant proportion of the carbon flux, 82% for heptanoate and 100% for octanol, flowed through the glyoxylate shunt. This indicates that the glyoxylate shunt plays a prominent role in the metabolism of heptanoate and octanol. Glucose catabolism was predicted to depend on the Entner–Doudoroff (ED) pathway because *P. putida* lacks 6-phosphofructokinase, which is a key enzyme in the Embden–Meyerhof–Parnas (EMP) pathway, leading to the utilization of the ED pathway instead [27, 54]. However, simulation of the catabolism of heptanoate, octanol, and styrene did not involve the ED pathway. Instead, it utilized the incomplete EMP pathway to a certain extent by generating glyceraldehyde 3-phosphate from isocitrate.

The predicted growth rate, ATP production, and NAD(P)H regeneration varied depending on the carbon source (Fig. 5B). The simulated growth rate was highest (0.99 h^−1^) when glucose was used as the sole carbon source, followed by octanol (0.70), heptanoate (0.67), and styrene (0.52). NADH regeneration was highest when octanol was used as the carbon source. This is because the metabolic pathway specific to octanol degradation involves several steps that produce NADH as a byproduct. Acetate was predicted to be produced as a byproduct during the catabolism of heptanoate and octanol, and not during the catabolism of glucose and styrene. Although GEMs inherently cannot take into account the solvent tolerance mechanisms of S12, including solvent efflux systems, toxin-antitoxin modules, and altered membrane composition [55, 56], these simulations suggest that iSH1474 can provide insights into the metabolic potentials and pathways associated with the catabolism of toxic organic solvents, which can contribute to a better understanding of the metabolism and potential applications of S12.

## Discussion

The metabolic versatility of *P. putida* strains varies in terms of their metabolic reaction content and substrate range [25]. As outlined in Fig. 1, the draft S12 metabolic model was constructed through a series of successive refinements beginning with mapping from GEMs of *P. putida* KT2440 (iJN1462) and *P. aeruginosa* PAO1 (iPAE1146) to the S12 genome, alongside incorporating the result from metabolic reconstruction using the RAVEN. This was followed by the adding of S12-specific reactions through manual effort. Phenotypic differences in S12 and KT2440 were assessed using PM data and the reported experimental data. This information was used to refine the metabolic model in order to ensure that it accurately represents the S12's metabolic capabilities. Therefore, both the draft model and the final iSH1474 differs largely from iJN1462 in terms of genetic and metabolic makeups. The differences in metabolic gene content between iSH1474 and iJN1462 were reflected in the results of gene essentiality analysis, which revealed S12- and KT2440-specific essential genes. To validate the model, its predictions were compared with various experimental data, including growth capabilities under different carbon sources, ^13^C-MFA data, and batch cultivation data (Fig. 4). This study highlights the importance of leveraging existing knowledge and comparative genome and phenome analyses in the reconstruction of strain-specific metabolic models.

iSH1474 was further evaluated using the metabolic model-testing suite MEMOTE [47], which assesses the quality and comprehensiveness of metabolic models. The overall score of iSH1474 was similar to that of the latest KT2440 GEM (Fig. 4B); however, there is still room for improvement compared to the most comprehensive GEMs of *E. coli* strains [57, 58]. Due to the insufficient external references for annotating genes, metabolites, and reactions of S12, the automatic reconstruction alone was insufficient. Therefore, extensive manual intervention was essential to overcome this limitation and enhance the quality of the model. Furthermore, a comparison of the model predictions with ^13^C-MFA data (Fig. 4C) showed that the S12 metabolism might be tightly regulated in response to environmental and cellular conditions. This suggests that integrating regulatory mechanisms, such as transcriptional regulation and signaling pathways, into the model would greatly enhance its accuracy and predictive capabilities.

Comparative genome and phenome analyses revealed that the observed differences in nutrient utilization between S12 and KT2440 (Fig. 3) may be associated with genetic differences (Fig. 2). Interestingly, only S12 harbors *dsdCXA,* which could explain the growth of S12 using D-serine as the sole carbon source. The role of *dsdCXA* has been demonstrated in detoxifying uropathogenic *E. coli* CFT073 from inhibitory levels of the host metabolite D-serine during infection [59]. The presence of *dsdCXA* in S12 could provide a selective advantage by allowing the strain to tolerate and utilize D-serine in environments with this metabolite.

S12 has excellent tolerance to various organic solvents [4, 6]. This unique property and its versatile metabolism make S12 a promising host for bioremediation and biotechnological applications. However, the development of bioprocesses utilizing S12 has been limited because of the lack of a comprehensive metabolic network model. As illustrated in Fig. 5, this model serves as a valuable tool for understanding the S12 metabolism and predicting its behavior under different conditions. In addition, iSH1474 has great potential for guiding experimental studies and identifying targets for metabolic engineering to improve industrial applications.

## Methods

### Identification of metabolic genes and reactions in *P. putida* S12

To identify the metabolic genes and reactions of S12 present in the published *Pseudomonas* GEMs, whole protein sequences of the genomes of *P. putida* KT2440 (RefSeq accession number: NC_002947.4) and *P. aeruginosa* PAO1 (NC_002516.2) were BLASTP-searched against those of the *P. putida* S12 chromosome (NZ_CP009974.1), using BLOSUM62 as a scoring matrix. As S12 is phylogenetically closely related to KT2440 than to PAO1, the initial homology searches for S12 genes were performed against the KT2440 genome. For any S12 genes without homologs identified in KT2440, a subsequent homology search was performed against the PAO1 genome. As a result, when S12 genes have homologs both in KT2440 and PAO1, the annotations from KT2440 were assigned to their corresponding S12 homologs. If the percentage of amino acid residues that are identical in the aligned region (percent identity) was over 90% and the aligned region was over 90% of the query length, the pair of sequences was considered a homolog.

To identify metabolic genes and reactions in S12 that are not present in iJN1462 and iPAE1146, the RAVEN 2.0 toolbox using the MetaCyc-based reconstruction module [32] was used for the automated metabolic reconstruction of S12. Genes with potential metabolic roles were further searched using BLASTP against the NCBI NR database with a cut-off query coverage of 90% and 40% identity, and their associated reactions were retrieved from the MetaCyc database. The maximum E-value for the search results was 2E-21.

### Calculation of GAM and NGAM

According to Pirt’s equation [60], the maximum growth yields and the maintenance coefficients can be calculated from linear regression: 1/*Y* = (*m*/μ) + (1/*Y*_G_), where *Y* is the observed growth yield, *Y*_G_ is the maximum growth yield, μ is the observed specific growth rate, and *m* is the maintenance coefficient which is the specific rate of substrate consumption required for maintenance purpose. Previously, Isken et al. [39] had determined the linear regression values, using data from glucose-limited chemostat cultures of *P. putida* S12 growing aerobically on a minimal glucose medium: 1/*Y*_protein_ = (0.023/*D*) + 3.00, where *Y*_protein_ is the protein yield (g protein/g glucose) and *D* is the dilution rate equivalent to the observed specific growth rate (μ). In this equation, the maintenance coefficient was 0.023 g glucose/g protein/h, and maximum protein yield was 1/3 g protein/g glucose. Considering that protein constitutes 60% of the total DCW in *P. putida* S12 [39], values of *m* and 1/*Y*_G_ were converted as in the units of g dry cell weight (DCW) and molar glucose. These converted values were used to generate another form of Pirt’s equation [60]: *q* = μ/*Y*_G_ + *m* or *q* = 10 μ + 0.077, where *q* is the specific rate of glucose consumption (mmol/gDCW/h). NGAM was predicted by running FBA simulation with iSH1474 by setting glucose uptake rate as 0.077 mmol glucose/gDCW/h and the reaction for ATP maintenance requirement (ATPM) as an objective function. This flux represents the ATP consumption even when the cell is not growing. The calculated NGAM (1.67 mmol ATP/gDCW/h) used to setting lower bounds of ATPM reaction in iSH1474. For GAM calculation, FBA was performed varying GAM values in the biomass equation to find the GAM value leading to the closest fit of the experimental plot of μ vs. *q* (Equation 3) (Fig. S1). The calculated GAM (42.31 mmol ATP/gDCW) was integrated to the model by biomass reaction.

### Phenotypic microarray test

*P. putida* strains S12 and KT2440 were purchased from the American Type Culture Collection. Preconfigured 96-well plates (Biolog Inc., Hayward, CA), known as PMs, contained various types of carbon (PM1 and PM2), nitrogen (PM3), phosphorus, sulfur (PM4), and auxotrophic supplements (PM5 to PM8). The PM9 and PM10 test stresses were molarity and pH, respectively. PM11 to PM20 contained inhibitory compounds, such as antibiotics, antimetabolites, and other inhibitors. Cells were cultured overnight at 37 °C on a BUG + B (Biolog universal growth medium + 5% sheep blood) agar plate. Colonies were picked from the agar surface and suspended in an inoculating fluid (IF) containing tetrazolium violet indicator dye. IF-0 media were used for plates PM1 to PM8 and IF-10 for plates PM9 to PM20. Sodium succinate was added with ferric citrate to the inoculation solutions of PM3–PM8. All PM plates were inoculated with cell suspensions at 100 µL/well and incubated at 37 °C for 48 h in an OmniLog incubator (Biolog Inc.). PM tests for each strain were performed in duplicate. PM data were analyzed using the opm package in R [61].

### Flux balance analysis

The FBA was performed as described previously [58] using the Python package COBRApy [37] and GLPK (https://www.gnu.org/software/glpk/) as the linear programming solver. The default objective function was the maximum growth rate of the biomass equation for iSH1474. To simulate aerobic growth, the upper limit of the oxygen uptake rate was set at 18.5 mmol/gDCW/h.

To simulate cell growth on the various carbon sources contained in PM1 and PM2, the composition of the defined medium used in the PM plates was retrieved from the BioCyc website [62] and used as the upper limit of the uptake rate of the corresponding component. The maximum uptake rate of each carbon source was set at 10 mmol/gDCW/h. A substrate was considered unutilized if the simulated growth rate was less than 5% of the growth objective value calculated for the cell growth on glucose. The flux distribution using heptanoate, octanol, styrene, or glucose as the sole carbon source was simulated using in silico M9 minimal medium with its maximum uptake rate of 10 mmol/gDCW/h.

To identify the essential genes, the maximum growth rates of single-gene deletions were simulated using an in silico M9 minimal medium [25] with a maximum glucose uptake rate of 6 mmol/gDCW/h. This was achieved by removing all the metabolic reactions associated with each gene from the metabolic model. A gene was considered essential if its removal from the model reduced the growth rate to less than 5% of the growth objective value calculated for the wild-type parental strain.

dFBA was performed using the “dynamicFBA” function in the COBRA toolbox v3.0 [63] running in MATLAB v9.6. The concentrations of glucose and biomass in the starting culture medium were set to 10 mM and 0.01 g/L, respectively. The maximum glucose uptake rate was set at 6.5 mmol/gDCW/h.

### Supplementary Information

**Additional file 1: Fig. S1.** Determination of GAM and NGAM in iSH1474 using chemostat data of *P. putida* S12. **Fig. S2. **Comparison of phenotype microarrays (PMs) of *P. putida* S12 with other strains. **Fig. S3.** Comparison of flux distribution in the central carbon metabolism of *P. putida* S12 from in vivo measurements and in silico predictions. **Fig. S4.** Comparison of predicted essential genes of *P. putida* strains S12 and KT2440 growing in a minimal glucose medium.

** Additional file 2: Table S1.** Metabolic reactions observed only in the GEM of *P. putida* S12 (iSH1474) compared to GEMs of *P. putida* KT2440 (iJN1462) and *P. aeruginosa* PAO1 (iPAE1146).

** Additional file 3. Table S2.** iSH1474 in Excel format.

** Additional file 4: Table S3.** Biomass equation of iSH1474.

** Additional file 5: Table S4.** Gene clusters observed only in the S12 genome (NZ_CP009974 and NZ_CP009975) and the KT2440 genome (NC_002947).

** Additional file 6: Table S5.** Phenotypic comparison of *P. putida* S12, *P. putida* KT2440, and *E. coli* BL21(DE3).

** Additional file 7: Table S6.** Comparison of simulated and experimental growth of *P. putida* S12 on various carbon sources.

** Additional file 8: Table S7.** Predicted essential genes of *P. putida* S12 using iSH1474 for aerobic growth on glucose as the sole carbon source.

** Additional file 9. File S1.** iSH1474 in SBML format.

## Data Availability

All data and codes for constructing the model and replicating the presented results are available at https://github.com/sybirg/s12_gem. The datasets supporting the conclusions of this article are included within the article and its additional files.
