# Supplementary material for: Genome-scale metabolic network model and phenome of solvent-tolerant Pseudomonas putida S12
Source: BMC Genomics. 2024 Jan 16;25:63. doi: 10.1186/s12864-023-09940-y (PMC10790481; doi:10.1186/s12864-023-09940-y)

**Fig. S1. Determination of GAM and NGAM in iSH1474 using chemostat data of *P. putida* S12.** The experimental plot of growth rate versus glucose uptake rate was generated using maintenance coefficient and maximum growth yield that were determined from glucose-limited chemostat cultures of *P. putida* S12 growing aerobically on a minimal medium (Isken *et al.*, *Applied and Environmental Microbiology*, 65:2631-35, 1999). The maximum oxygen uptake rate for flux balance analysis was set to 18.5 mmol/gDCW/h.


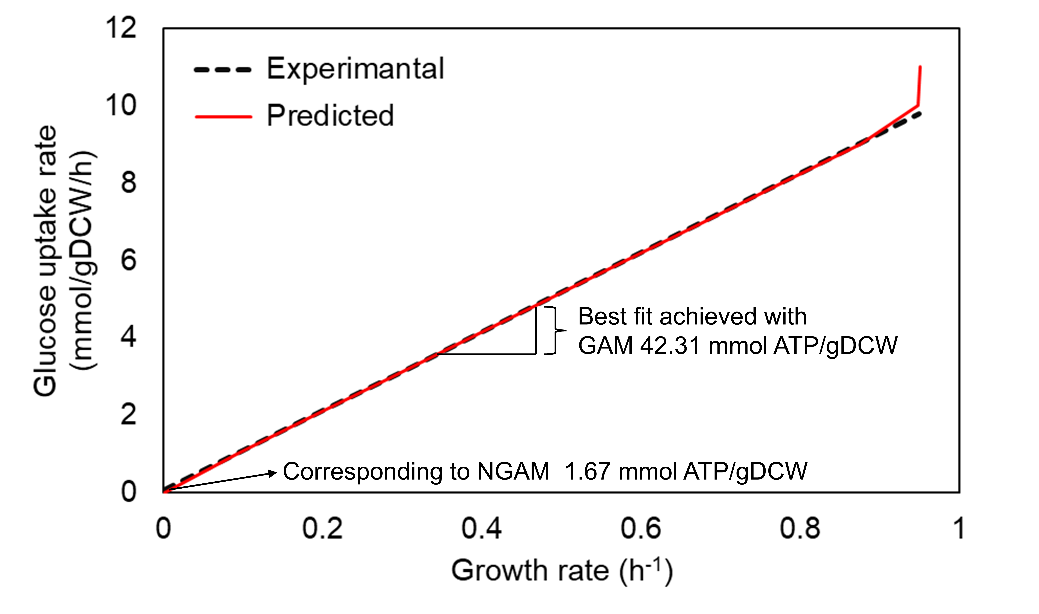


**Fig. S2. Comparison of phenotype microarrays (PMs) of *P. putida* S12 with other strains. (A)** *P. putida* S12 vs. *P. putida* KT2440. (**B)** *P. putida* S12 vs. *E. coli* BL21(DE3). Growth curves during 48 h are colored red for faster growth of S12, green for faster growth of KT2440 or BL21(DE3), and yellow for similar growth of both strains. The PM plates consisted of 20 96-well microplates containing different sources of carbon (PM1 and PM2), nitrogen (PM3), phosphorus, sulfur (PM4), auxotrophic supplements (PM5), peptide nitrogen sources (PM6 to PM8), osmolytes (PM9), pH stress (PM10), and inhibitory compounds such as antibiotics, antimetabolites, and other inhibitors (PM11 to PM20). Detailed information on PM plates can be obtained at [www.biolog.com](http://www.biolog.com).


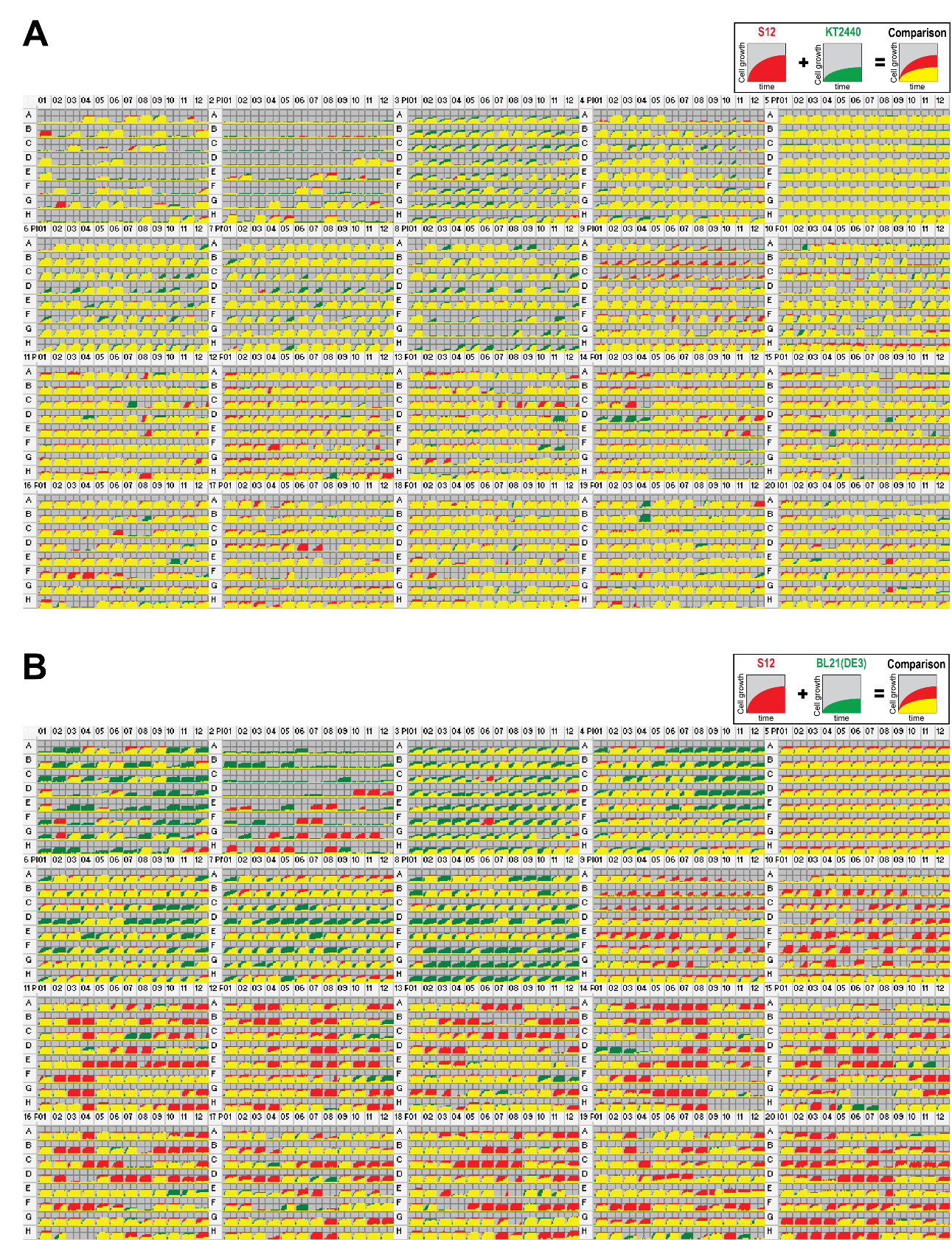


**Fig. S3. Comparison of** **flux distribution in the central carbon metabolism of *P. putida* S12 from *in vivo* measurements and *in silico* predictions.** The flux simulations using iSH1474 (S12) or iJN1462 (KT2440) were compared with the fluxes from ^13^C-based metabolic flux analysis of S12 (Blank et al. *The FEBS Journal*, 275:5173-5190, 2008). All flux values are given in mmol/gDCW/h. The box shows the experimental flux values (top) and the flux simulations using iJN1462 (middle) or iJN1462 (bottom). Metabolites and metabolic reactions are colored in grey and black, respectively. Arrows denote the directionality of the reactions, with black arrow heads being the forward reactions and grey heads being the reverse reactions. Abbreviations are given in Table S2.


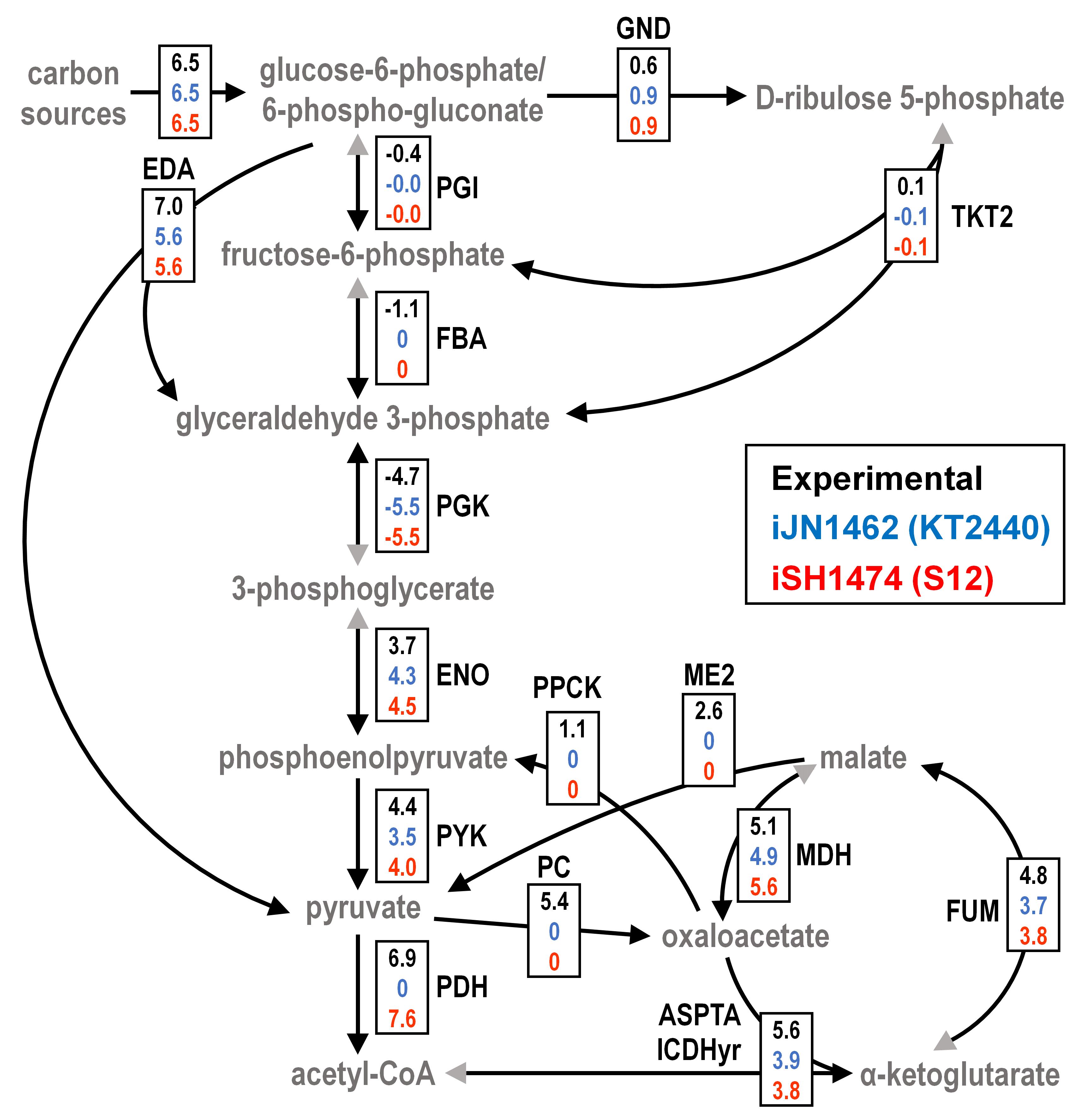


**Fig. S4. Comparison of predicted essential genes of *P. putida*** **strains S12 and KT2440 growing in a minimal glucose medium.** Metabolic models of S12 (iSH1474) and KT2440 (iJN1462) were used for single gene deletion simulations. **(A)** Venn diagram showing the number of predicted essential genes for S12 and KT2440. (**B**) Predicted essential genes specific to S12 and KT2440. A complete list of the predicted essential genes of S12 is available in Table S7, and detailed information on reactions can be obtained in Table S2.


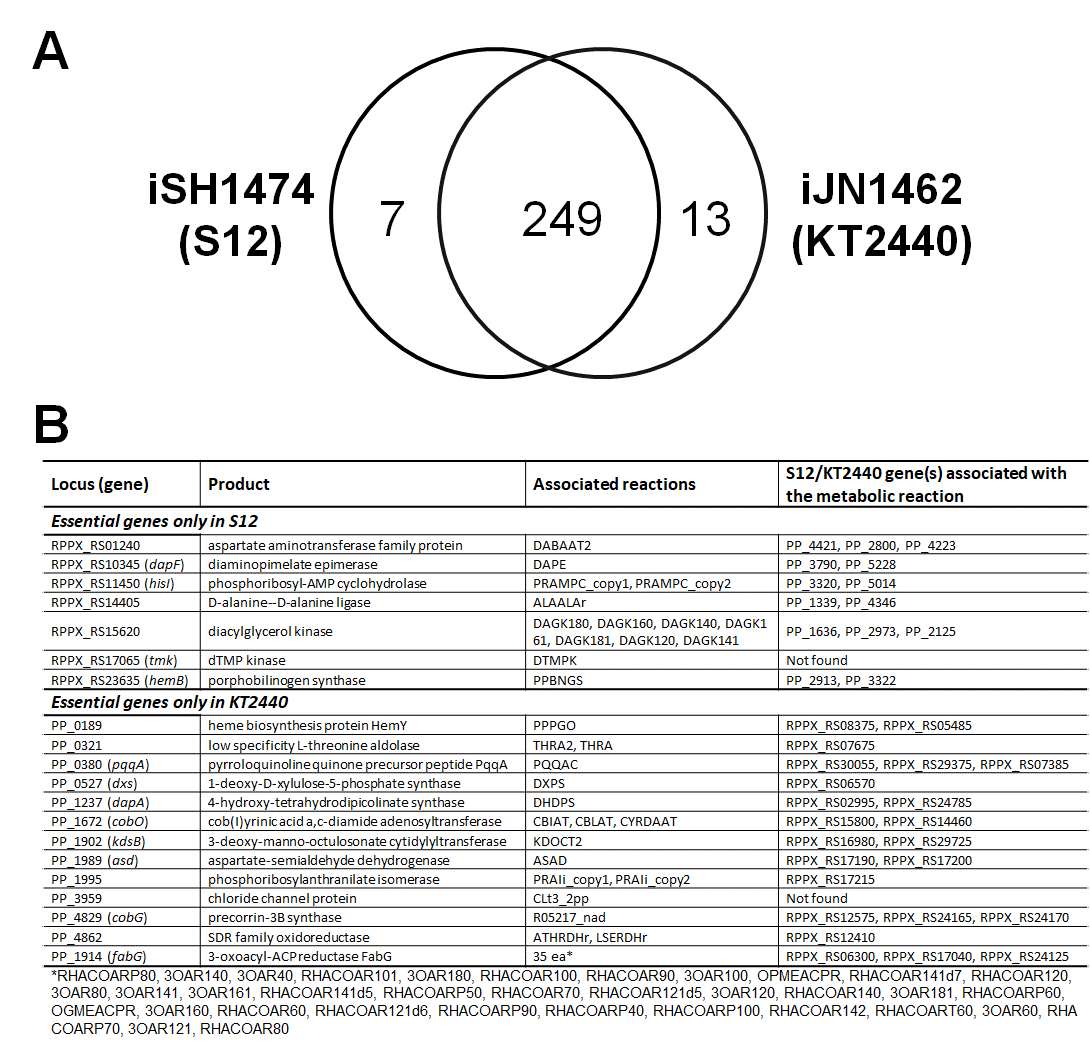

Supplement: Supplementary file 1 — Additional file 1: Fig. S1. Determination of GAM and NGAM in iSH1474 using chemostat data of P. putida S12. Fig. S2. Comparison of phenotype microarrays (PMs) of P. putida S12 with other strains. Fig. S3. Comparison of flux distribution in the central carbon metabolism of P. putida S12 from in vivo measurements and in silico predictions. Fig. S4. Comparison of predicted essential genes of P. putida strains S12 and KT2440 growing in a minimal glucose medium. [file 12864_2023_9940_MOESM1_ESM.docx]
